# MicroRNA-98 reduces nerve growth factor expression in nicotine-induced airway remodeling

**DOI:** 10.1074/jbc.RA119.012019

**Published:** 2025-01-13

**Authors:** Cherry Wongtrakool, Junsuk Ko, Andrew J. Jang, Kora Grooms, Sarah Chang, Cory Sylber, Beata Kosmider, Karim Bahmed, Michael R. Blackburn, Roy L. Sutliff, C. Michael Hart, Changwon Park, Toru Nyunoya, Michael J. Passineau, Qing Lu, Bum-Yong Kang

**Affiliations:** 1 Department of Medicine, Atlanta Veterans Affairs Healthcare System and Emory University School of Medicine, Atlanta, Georgia, USA; 2 Department of Biochemistry and Molecular Biology, University of Texas Health Science Center, Houston, Texas, USA; 3 Cardiovascular Institute, Department of Medicine, Allegheny Health Network, Pittsburgh, Pennsylvania, USA; 4 Center for Inflammation, Translational and Clinical Lung Research, Department of Thoracic Medicine and Surgery, and Department of Physiology, Temple University School of Medicine, Philadelphia, Pennsylvania, USA; 5 Department of Cellular and Molecular Physiology, Louisiana State University Health Science Center, Shreveport, Louisiana, USA; 6 Department of Medicine, University of Pittsburgh, Pittsburgh, Pennsylvania, USA; 7 Vascular Research Laboratory, Providence Veterans Affairs Medical Center/Alpert Medical School of Brown University, Providence, Rhode Island, USA

**Keywords:** lung, neurotrophin, fibroblast, microRNA, asthma, nerve growth factor, miR-98, airway hyperresponsiveness, nicotine, airway remodeling, microRNA (miRNA)

## Abstract

Evolving evidence suggests that nicotine may contribute to impaired asthma control by stimulating expression of nerve growth factor (NGF), a neurotrophin associated with airway remodeling and airway hyperresponsiveness. We explored the hypothesis that nicotine increases NGF by reducing lung fibroblast (LF) microRNA-98 (miR-98) and PPARγ levels, thus promoting airway remodeling. Levels of NGF, miR-98, PPARγ, fibronectin 1 (FN1), endothelin-1 (EDN1, herein referred to as ET-1), and collagen (COL1A1 and COL3A1) were measured in human LFs isolated from smoking donors, in mouse primary LFs exposed to nicotine (50 μg/ml), and in whole lung homogenates from mice chronically exposed to nicotine (100 μg/ml) in the drinking water. In selected studies, these pathways were manipulated in LFs with miR-98 inhibitor (anti-miR-98), miR-98 overexpression (miR-98 mimic), or the PPARγ agonist rosiglitazone. Compared with unexposed controls, nicotine increased NGF, FN1, ET-1, COL1A1, and COL3A1 expression in human and mouse LFs and mouse lung homogenates. In contrast, nicotine reduced miR-98 levels in LFs *in vitro* and in lung homogenates *in vivo*. Treatment with anti-miR-98 alone was sufficient to recapitulate increases in NGF, FN1, and ET-1, whereas treatment with a miR-98 mimic significantly suppressed luciferase expression in cells transfected with a luciferase reporter linked to the putative seed sequence in the NGF 3′UTR and also abrogated nicotine-induced increases in NGF, FN1, and ET-1 in LFs. Similarly, rosiglitazone increased miR-98 and reversed nicotine-induced increases in NGF, FN1, and ET-1. Taken together, these findings demonstrate that nicotine-induced increases in NGF and other markers of airway remodeling are negatively regulated by miR-98.

Cigarette smoke exposure is associated with airway remodeling and airway hyperresponsiveness (AHR) in humans and in a variety of experimental models (1, 2, 3, 4). Persistent AHR is also associated with poorer asthma and increased symptoms (5, 6). The complex pathophysiology of AHR involves inflammation, airway smooth muscle cell (SMC) dysfunction, neuronal signaling, and airway remodeling (7, 8). Airway remodeling involves structural changes in the airway wall, including increased airway smooth muscle mass, angiogenesis, mucous gland hypertrophy, epithelial damage, change in extracellular matrix composition, and subepithelial fibrosis (9). Fibroblast activation is necessary for the development of airway remodeling and subsequent AHR (10). Previous studies demonstrate that the offspring of mothers exposed to nicotine during the prenatal period have increased AHR in the postnatal period that persists into adulthood and that is associated with increased collagen deposition in the airway wall (11, 12). However, the effects on chronic nicotine exposure in the adult on airway remodeling remain unclear. The molecular mechanisms of cigarette smoke–induced airway remodeling are not fully known, but nicotine, the addictive component of cigarette smoke, has been implicated as a mediator of airway remodeling (13). Given the rapidly growing use of electronic nicotine delivery devices, particularly in teens and youths, it is important to understand the role of nicotine in promoting airway remodeling as a contributing factor to AHR. This study focuses on a novel mechanism by which nicotine stimulates airway remodeling through nerve growth factor (NGF) and contributes to the development of AHR.

NGF, a neurotrophin responsible for neuronal growth and differentiation, fosters the development of AHR through Immunoglobulin E–mediated inflammation, tachykinin signaling, cholinergic nerve sensitization, and airway remodeling (14). Airway remodeling occurs in animal models of exogenous administration or transgenic overexpression of NGF (15, 16). NGF overexpression increases type III collagen deposition by airway fibroblasts through transforming growth factor β1–independent signaling (17). Although multiple cell types in the lung produce NGF, including macrophages, lymphocytes, and epithelial cells, fibroblasts play an important role in airway remodeling (18, 19, 20, 21). Kohan *et al.* (10) reported in an ovalbumin model of asthma that decreased fibroblast activation was associated with reduced subepithelial fibrosis, collagen deposition, and AHR in the absence of any reduction in airway inflammation. Prior studies demonstrate that chronic nicotine exposure increases NGF levels in the lung through nicotine activation of the α7 nicotinic acetylcholine receptor (21). Nicotine-exposed lung fibroblasts (LFs) produce NGF and, when cocultured with airway smooth muscle cells, stimulate NGF-dependent smooth muscle cell contractile protein expression, suggesting that paracrine signaling from fibroblasts residing in the airway wall influences airway contractile function and thus AHR (21). Therefore, the current study extends these observations to further investigate how NGF production by LF in response to chronic nicotine exposure contributes to airway remodeling and further clarifies how NGF expression is regulated in the lung (22).

The current study provides evidence that nicotine modulates levels of microRNAs (miRs or miRNAs), small noncoding RNA strands that negatively regulate gene expression through mRNA degradation or translational repression. Recent studies implicate the let-7 family in the pathogenesis of AHR and asthma (23, 24). Members of the miR-98/let-7 family are downregulated in lung and other cancers and regulate apoptosis and inflammation (25, 26, 27, 28, 29). For example, let-7 levels are decreased in the bronchoalveolar lavage fluid exosomes of asthmatics compared with healthy controls, suggesting that let-7 may have important regulatory functions in asthma and/or AHR (23, 24). Interestingly, let-7 family members are significantly downregulated in the lungs of mice exposed to cigarette smoke (30). However, the precise mRNA targets regulated by miR-98/let-7 family and their contribution to the pathogenesis of AHR and asthma in nicotine-associated airway remodeling have not been defined. In other studies, miR-98/let-7 family members inhibit cell growth responses and function as tumor suppressors in lung cancer, in part through regulation of apoptosis and inflammation (25, 26, 27, 28, 29, 31).

The study findings define a novel regulatory role for a peroxisome proliferator–activated receptor γ (PPARγ)–miR-98 axis in nicotine-induced NGF expression and airway remodeling in LFs associated with AHR by demonstrating that PPARγ activation restores miR-98 levels to suppress nicotine-induced NGF, ET-1, and FN1. These findings suggest that targeting these miRNAs involved in airway remodeling and asthma pathogenesis may yield new therapeutic opportunities in asthma management, possibly by activating PPARγ in LFs.

## Results

### Chronic nicotine exposure increases AHR

**Figure 1:**
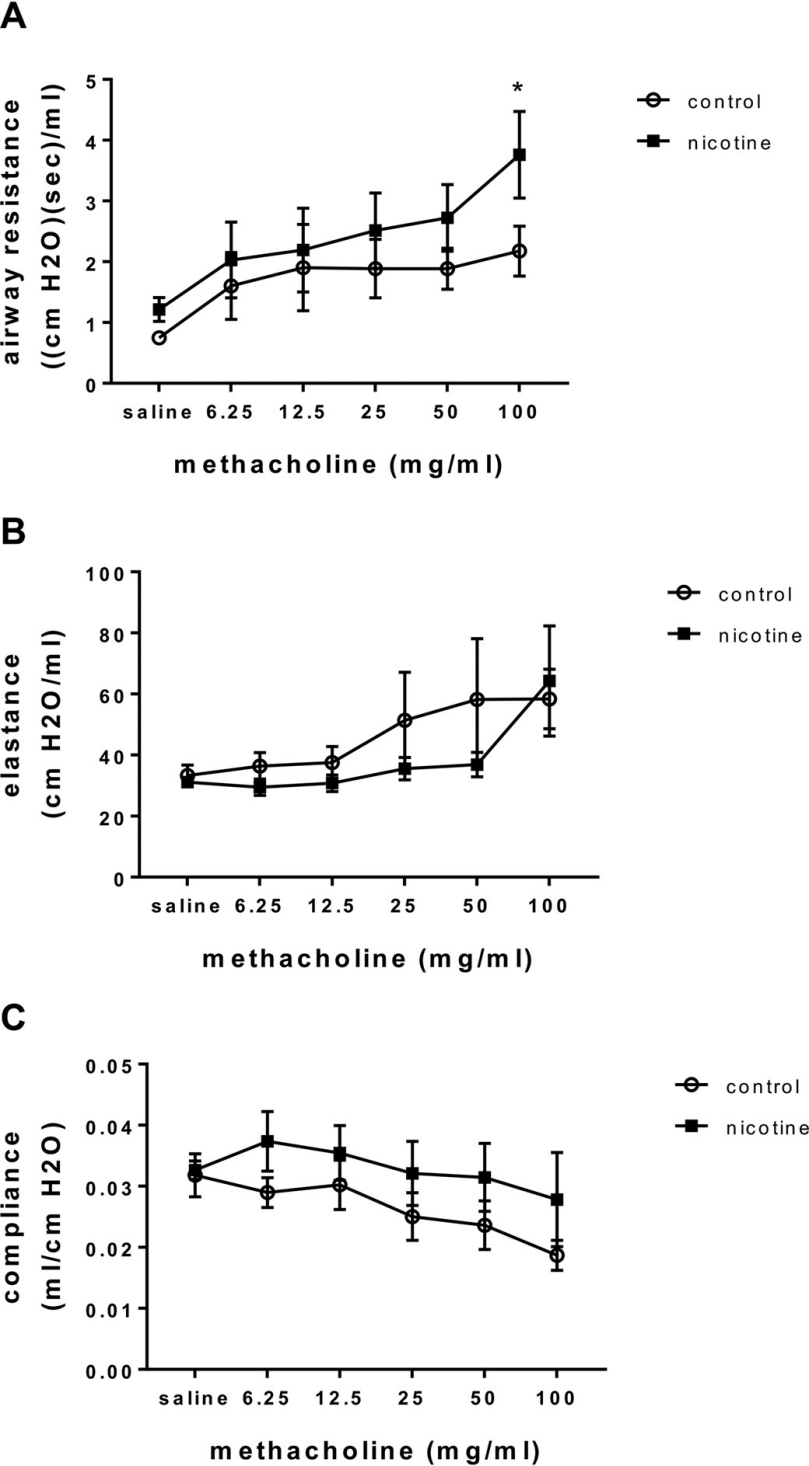
**Chronic nicotine exposure increases AHR.** C57Bl/6J mice were administered nicotine (100 µg/ml) in the drinking water *ad libitum* for 4 weeks. Animals were anesthetized, tracheostomized, and mechanically ventilated for pulmonary function testing. Airway resistance (*A*), lung elastance (*B*), and lung compliance (*C*) were measured at baseline after inhaled saline and after increasing doses of methacholine using a flexiVent (EMKA). *n* = 3–5, *p < 0.05 when compared with untreated controls.

Although previous work demonstrates that offspring of maternal dams exposed to nicotine *in utero* have increased AHR, the effects of chronic nicotine exposure during adulthood on AHR are poorly defined (32). To further examine the effects of chronic nicotine exposure on AHR, adult C57BL/6J mice were administered nicotine in the drinking water *ad libitum* for 4 weeks as we previously reported (21). This model of oral nicotine delivery has been shown to produce plasma nicotine levels similar to smoking a pack of cigarettes per day in humans and is similar to other mouse models delivering nicotine via aerosol or subcutaneous injection (33, 34). Delivering nicotine orally minimizes stress from handling of the animals, and the animals' ability to drink *ad libitum* allows them to mimic a smoker's pattern of using cigarettes and subsequently nicotine exposure. Airway hyperresponsiveness was assessed by measuring airway resistance using the forced oscillation technique at baseline and with increasing doses of inhaled methacholine. Animals chronically exposed to nicotine exhibited increased AHR as defined by increased airway resistance in response to methacholine when compared with untreated controls even in the absence of allergic sensitization (Fig. 1*A*). Elastance and compliance were not significantly different when compared with untreated controls. (Fig. 1, *B* and *C*).

### Nicotine increases NGF levels and reduces miR-98 expression

**Figure 2:**
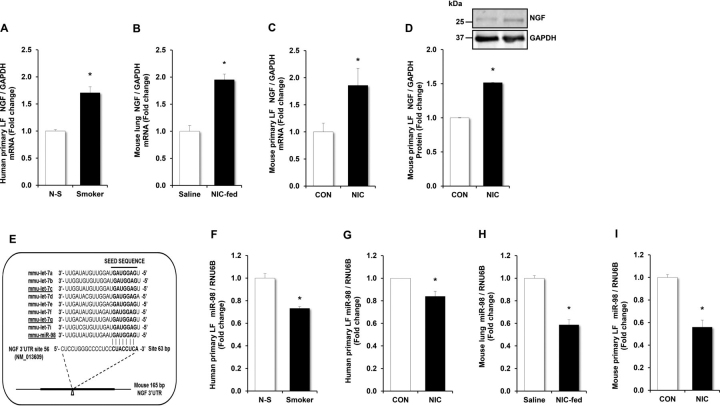
**Nicotine exposure increases NGF and decreases miR-98.** Human primary LFs obtained from smoking (*Smoker*, *n* = 4) and nonsmoking (*N-S*, *n* = 4) lung donors were cultured for 72 h. mRNA or miRNA were isolated and subjected to qRT-PCR analysis for NGF and miR-98, using GAPDH or RNU6B as controls. Each bar represents the mean ± S.E. (*error bars*) of NGF (*A*) or miR-98 (*F*) relative to GAPDH or RNU6B, expressed as fold change. Human primary LFs were treated with nicotine (50 µg/ml) or an equivalent volume of vehicle in culture medium (*CON*) for 72 h. Human LF miRNA was then isolated. miR-98 levels were measured with qRT-PCR relative to RNU6B, expressed as fold change (*G*). *p < 0.05 *versus N-S*. *n* = 3. Whole lung homogenates obtained from mice chronically fed with nicotine (*NIC-fed*, 100 µg/ml) or saline in the drinking water for 4 weeks. qRT-PCR was performed on mouse lung tissue to assess expression of NGF (*B*) and miR-98 (*H*). Each bar represents the mean ± S.E. (*error bars*) of NGF or miR-98 relative to GAPDH or RNU6B and normalized to control values. *p < 0.05 *versus Saline*, *n* = 4–5. *In silico*–predicted miRNAs having potential binding sites in the NGF 3′UTR. The mouse NGF 3′UTR (NM_013609) contains a putative binding site (*arrowhead*) for let-7/miR-98 family. The let-7/miR-98 seed sequence is shown in *bold font*. The mouse 165-bp NGF mRNA 3′UTR containing a putative miR-98 binding site was confirmed on agarose gel (*E*). Mouse primary LFs isolated from C57BL/6 were treated with nicotine (50 µg/ml) or an equivalent volume of vehicle in culture medium (*CON*) for 72 h. Mouse LF mRNA, miRNA, or protein were then isolated. NGF or miR-98 levels were measured with qRT-PCR and/or Western blotting. Each bar represents the mean ± S.E. (*error bars*) of NGF (*C*) or miR-98 (*I*) relative to GAPDH or RNU6B in the same sample expressed as fold change *versus* CON. *n* = 6, *p < 0.05 *versus* CON. Western blotting was employed to detect NGF and GAPDH protein levels (*D*). Representative blot depicting NGF and GAPDH protein in nicotine-treated LFs is shown. Mouse primary LFs were treated with nicotine (50 µg/ml) or an equivalent volume of vehicle in culture medium (*CON*) for 72 h. Mouse LF miRNA was then isolated. miR-98 levels were measured with qRT-PCR relative to RNU6B, expressed as fold change (*I*).

Animals overexpressing NGF have increased AHR and changes of airway remodeling (35). Prior work shows that nicotine stimulates NGF secretion by murine LFs, cells that play a key role in airway remodeling (21). To determine whether similar alterations were present in the airways of human smokers, mRNA expression of NGF was examined in LFs harvested and cultured from donor lungs from human smokers and nonsmokers. Levels of NGF mRNA remained persistently and significantly increased in LFs from smokers (Fig. 2*A*). Similarly, chronic nicotine exposure increased NGF mRNA levels in whole lungs harvested from animals exposed to nicotine chronically for 4 weeks (Fig. 2*B*) and increased NGF mRNA and cellular protein levels *in vitro* in mouse LFs exposed to nicotine for 72 h (Fig. 2, *C* and *D*). Further nicotine increased NGF levels in human pulmonary artery smooth muscle cells, human pulmonary artery endothelial cells, and human bronchial epithelial cell 2 (HBEC2) (Fig. S1).

Because miRNAs are potential regulators of NGF expression and the let-7 family of miRNAs contribute to the pathogenesis of asthma, NGF-targeted miRNAs were identified with bioinformatic resources of microRNA target predictions (www.mirdb.org, miRDB and TargetScan v7.1) (23, 24). These analyses confirmed that the NGF 3′UTR contains binding sites for the seed sequences of miR-98/let-7 families which are highly conserved in human and mouse (Fig. 2*E*). We then screened for nicotine-induced alterations in the levels of miR-98/let-7 families. miR-98, let-7a, and let-7g were significantly and consistently downregulated in human LFs obtained from smokers, lung tissue from mice fed nicotine *in vivo*, and mouse LFs exposed to nicotine *in vitro* (Fig. 2, *F*–*I* and Fig. S2). Furthermore, nicotine decreased miR-98 expression in a dose- and time-dependent manner in mouse LFs (Fig. S3). Because miR-98 is a known regulator of PPARγ, a transcription factor that plays a key role in mediating airway remodeling in prenatal nicotine exposure, we focused on examining the potential role of miR-98 in regulating NGF in airway remodeling and asthma therapy (30, 36).

### miR-98 regulates nicotine-stimulated NGF levels

**Figure 3:**
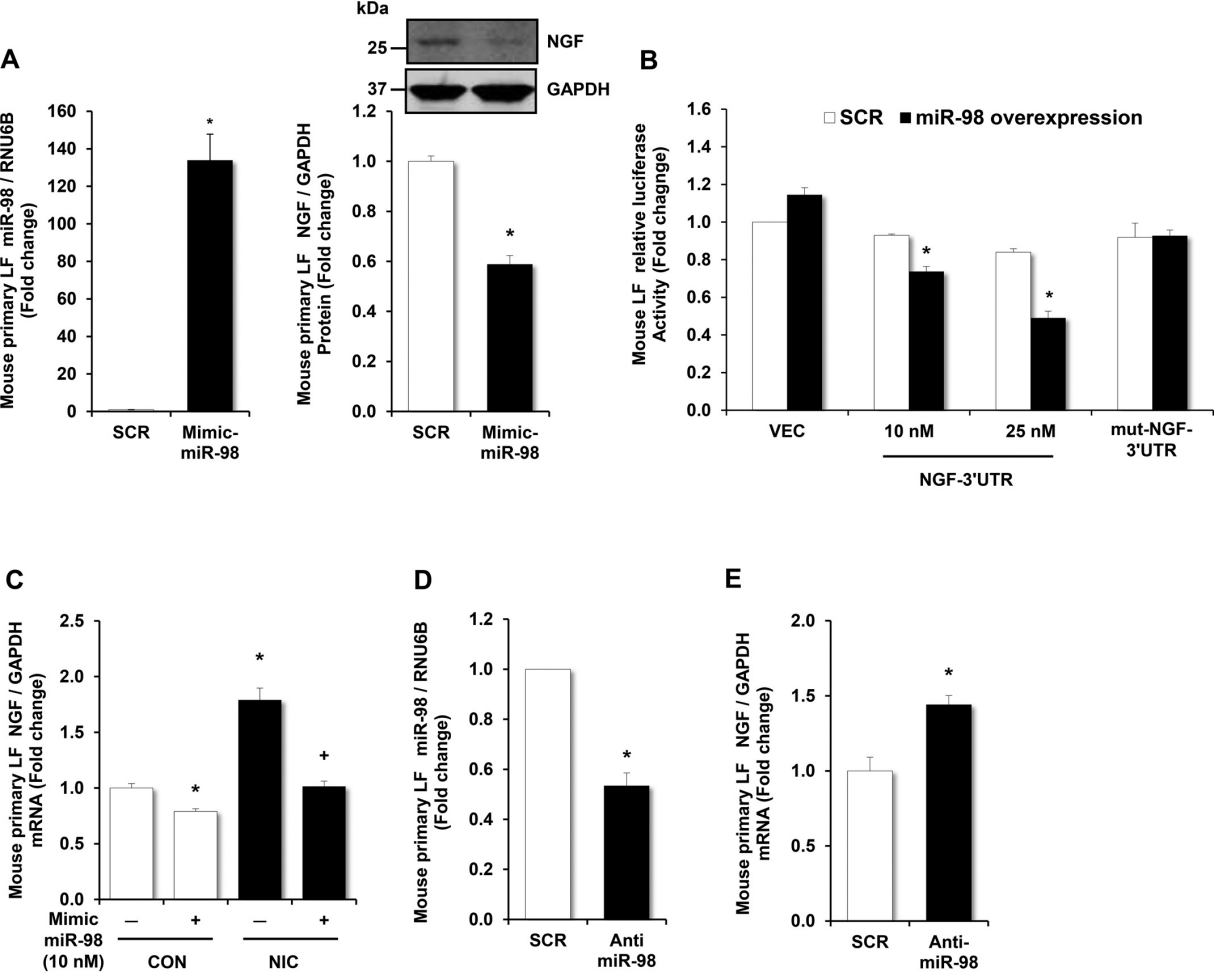
**miR-98 negatively regulates nicotine-stimulated NGF expression.***A*, mouse LFs were transfected with scrambled mimic (10 nm) or miR-98 mimic (10 nm) for 6 h, and then mouse LFs were incubated for 72 h. After 72 h, mouse LF miRNA or protein were then isolated. miR-98 or NGF levels were measured with qRT-PCR or Western blotting. Each bar represents the mean ± S.E. (*error bars*) of miR-98 or NGF. *B*, mouse primary LFs were transfected with luciferase reporter plasmids containing the putative seed sequence in the 3′UTR region of NGF mRNA or a mutated seed sequence. After transfection, mouse LFs were treated with a miR-98 mimic (10 nm and 25 nm) that specifically up-regulated miR-98 expression for 72 h, and luciferase activity was measured. *Error bars* represent mean ± S.E., *n* = 3, *p < 0.05 *versus* SCR-NGF-3′UTR constructs. *C*, mouse LFs were transfected with scrambled mimic (10 nm) or miR-98 mimic (10 nm) for 6 h and then treated with or without nicotine (*NIC*, 50 µg/ml) for 72 h. mRNA was isolated for qRT-PCR analysis. *Error bars* represent mean ± S.E., *n* = 11, *p < 0.05 *versus* untreated controls (*CON*), +*p* < 0.05 *versus* NIC. *D* and *E*, mouse LFs were treated with either scrambled miR (*SCR*) or 50 nm anti-miR-98 for 6 h and then treated with nicotine (50 µg/ml) or an equivalent volume of culture medium (*CON*) for 72 h. Mouse LFs were then collected, and miRNA or mRNA were isolated and subjected to qRT-PCR analysis for miR-98, NGF, RNU6B, or GAPDH. Each bar represents the mean ± S.E. (*error bar*) of miR-98 (*D*) or NGF (*E*) relative to RNU6B or GAPDH expressed as fold change *versus* SCR. *p < 0.05 *versus* SCR. *n* = 5.

Hypothesizing that miR-98 directly regulates NGF in lung fibroblasts, we verified that miR-98 overexpression significantly decreased NGF mRNA stability (Fig. S4). To further confirm that NGF is a miR-98 target, we performed a luciferase assay with a plasmid containing a luciferase reporter linked to the putative seed sequence in the 3′UTR region of NGF mRNA. As illustrated in Fig. 3*A*, transfection of a miR-98 mimic significantly and specifically increased miR-98 levels and decreased NGF protein levels but did not change let-7c and let-7g levels (Fig. S5) when compared with cells treated with a scrambled miRNA (SCR). The overexpression of miR-98 significantly decreased luciferase activity in a dose-dependent manner in cells transfected with the WT NGF 3′UTR luciferase reporter, whereas luciferase activity was unchanged in cells transfected with the mutant plasmid (Fig. 3*B*). In mouse LFs exposed to nicotine, treatment with the miR-98 mimic abrogated the nicotine-stimulated increases in NGF (Fig. 3*C*). Similarly, anti-miR-98 treatment reduced fibroblast miR-98 levels comparable in magnitude to those caused by exposure to nicotine (Fig. 3*D*) and recapitulated the nicotine-induced increases in NGF mRNA levels (Fig. 3*E*). Because miRNAs activate the RNA-induced silencing complex to silence complementary target mRNAs, we performed an RNA-binding protein immunoprecipitation (RIP) assay using anti-Ago2 antibody, which is the key component of the functional RNA-induced silencing complex that cleaves the target mRNA strand (37). Anti-Ago2 RNA immunoprecipitation experiments show a significant enrichment in miR-98 when compared with IgG immunoprecipitates as the input control and an enrichment in NGF in the presence of miR-98 mimic (Fig. S6). These results suggest that miR-98 directly targets and negatively regulates expression of nicotine-induced NGF.

### Nicotine increases expression of additional airway remodeling markers through NGF

**Figure 4:**
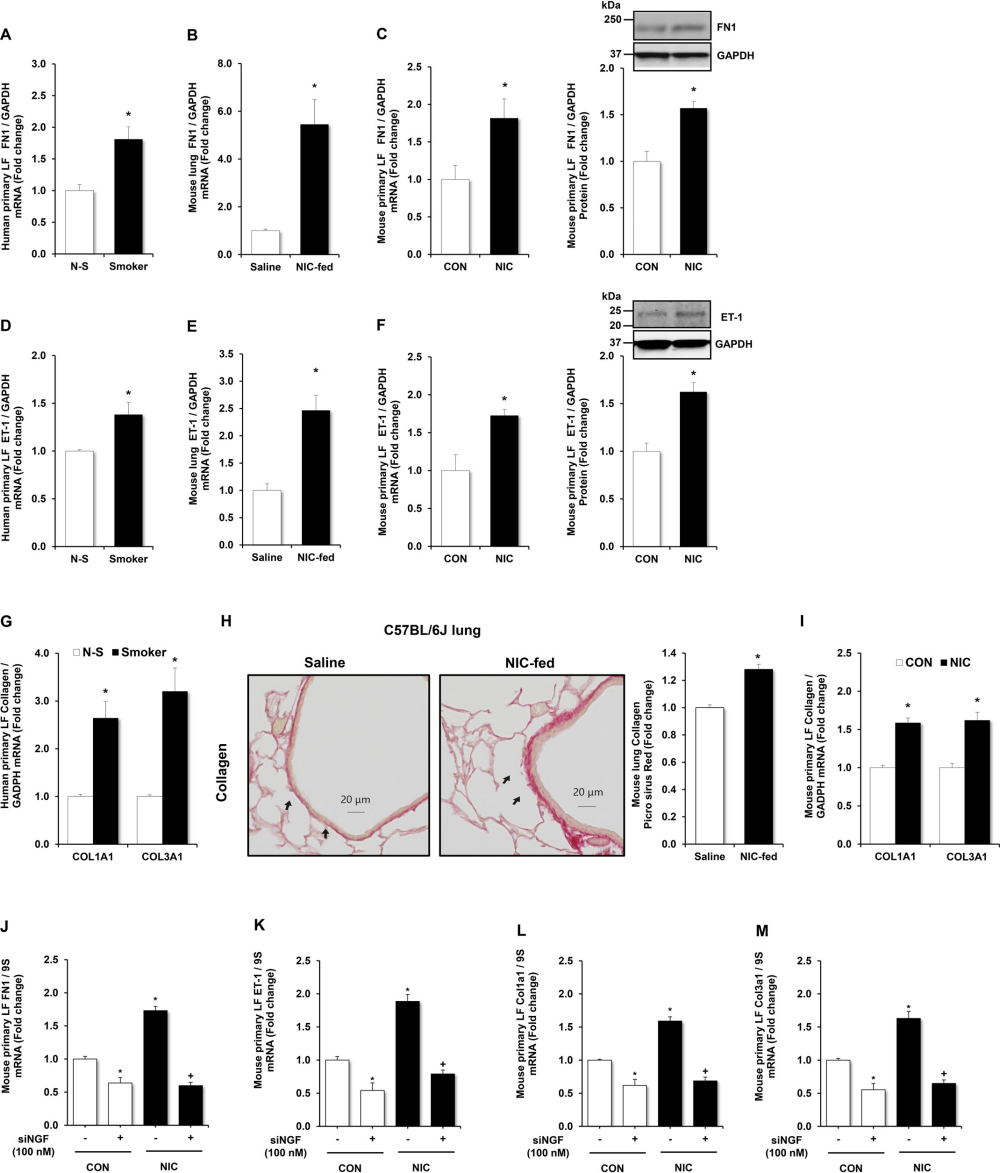
**Nicotine increases expression of airway remodeling markers.** Human primary lung fibroblasts (LFs) obtained from smoking (*Smoker*) and nonsmoking (*N-S*) lung donors were cultured for 72 h. mRNAs were isolated and subjected to qRT-PCR analysis for FN1, ET-1, COL1A1, and COL3A1, using GAPDH as control. Each bar represents the mean ± S.E. (*error bars*) FN1 (*A*), ET-1 (*D*), or COL1A1 and COL3A1 (*G*) relative to GAPDH expressed as fold change *versus N-S*. *p < 0.05 *versus N-S*. *n* = 3. Whole lung homogenates obtained from mice chronically fed with nicotine (*NIC-fed*, 100 µg/ml) or saline (*Saline*) in the drinking water for 4 weeks. qRT-PCR was performed on mouse lung tissue for FN1 (*B*), ET-1 (*E*) expression, or peribronchial collagen (*H*) deposition. NIC-exposed lungs had increased peribronchial collagen deposition as examined by Picrosirius Red staining. Red staining indicated by the *arrowheads* is collagen deposition (40×). Each bar represents the mean ± S.E. (*error bars*) FN1 or ET-1 relative to GAPDH and normalized to control values. *p < 0.05 *versus Saline*, *n* = 4–5. Mouse primary LFs isolated from C57BL/6 were treated with nicotine (50 µg/ml) or an equivalent volume of vehicle in culture medium (*CON*) for 72 h. Mouse LF mRNA or protein were then isolated. Mean mouse LF FN1, ET-1, or COL1A1 and COL3A1 levels were measured with qRT-PCR and/or Western blotting. Each bar represents the mean ± S.E. (*error bars*) FN1 (*C*), ET-1 (*F*), or COL1A1 and COL3A1 (*I*) relative to GAPDH mRNA in the same sample expressed as fold change *versus* CON. *n* = 6, *p < 0.05 *versus* CON. Western blotting was employed to detect FN1, ET-1, or GAPDH protein levels. Representative blots depicting FN1, ET-1, or GAPDH protein in nicotine-treated LFs is shown. Mouse LFs were transfected with siNGF (100 nm), then treated with or without nicotine (*NIC*, 50 µg/ml) for 72 h. Mouse LF mRNA was then isolated. Treatment with siNGF abrogated nicotine (*NIC*)–induced increases in FN1 (*J*), ET-1 (*K*), COL1A1 (*L*), and COL3A1 (*M*) mRNA levels. *n* = 3, *p < 0.05 *versus* CON.

Cigarette smoke exposure has been closely associated with airway remodeling. Prior studies in the developing lung demonstrate that nicotine plays a key role in mediating airway remodeling via α7 nicotinic acetylcholine receptor-mediated signaling (12). However, the mechanisms underlying the interaction between nicotine exposure, airway remodeling, and AHR have not been fully explored in the nondeveloping lung. Thus, to determine whether nicotine can induce airway remodeling associated with AHR, we measured the expression of ET-1, FN1, and collagen markers of airway remodeling in primary lung fibroblasts. Levels of FN1, ET-1, COL1A1, and COL3A1 mRNAs were significantly increased in LFs from smokers (Fig. 4, *A*, *D*, and *G*).

Similarly, chronic nicotine exposure increased mouse lung FN1 and ET-1 levels and peribronchial collagen deposition (Fig. 4, *B*, *E*, and *H*). Exposing mouse LFs to nicotine *in vitro* for 72 h increased FN1, ET-1, COL1A1, and COL3A1 mRNA and protein levels (Fig. 4, *C*, *F*, and *I*). However, NGF siRNA knockdown abrogates nicotine-induced increases in FN1, ET-1, COL1A1, and COL3A1 mRNA expression (Fig. 4, *J*–*M* and Fig. S7). Collectively, these findings suggest that nicotine treatment can induce airway remodeling in AHR and that nicotine induction of airway remodeling markers is mediated by NGF.

### miR-98 loss- or gain-of-function alters FN1 and ET-1 expression

**Figure 5:**
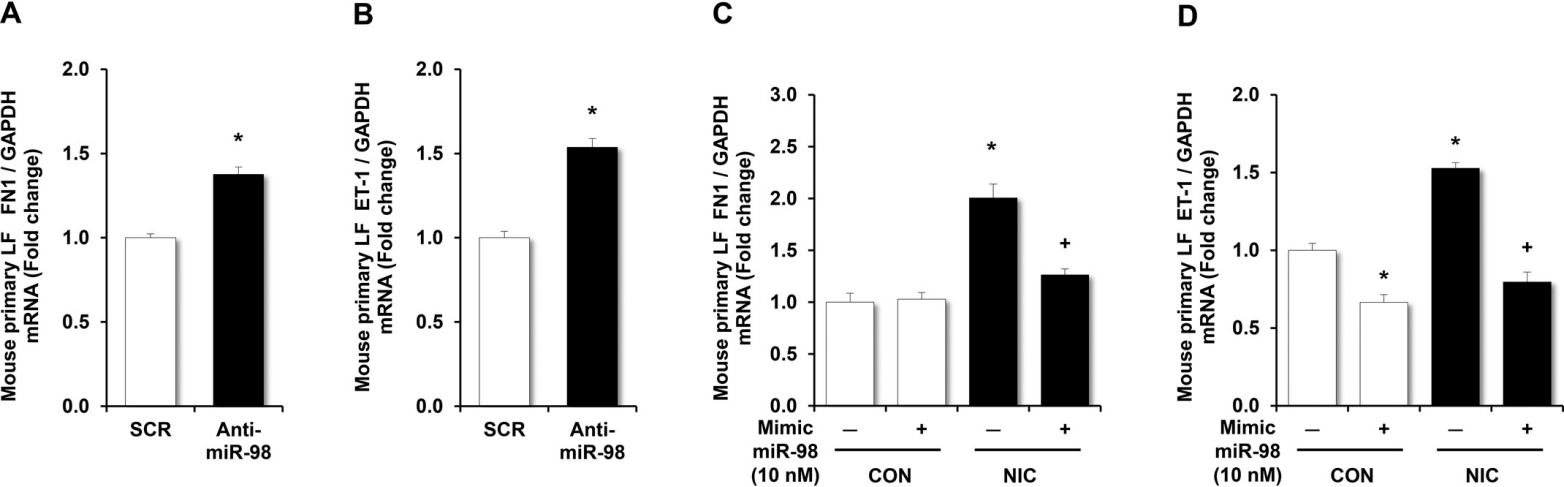
**miR-98 loss- or gain-of-function regulates FN1 and ET-1 expression.***A* and *B*, mouse LFs were treated with either scrambled miR (*SCR*) or 50 nm anti-miR-98 for 6 h and then treated with nicotine (50 µg/ml) or an equivalent volume of vehicle in culture medium (*CON*) for 72 h. Mouse LFs were then collected, and mRNA were isolated and subjected to qRT-PCR analysis for FN1, ET-1, or GAPDH. Each bar represents the mean ± S.E. (*error bars*) FN1 (*A*) or ET-1 (*B*) relative to GAPDH expressed as fold change *versus* SCR. *p < 0.05 *versus* SCR. *n* = 5. *C* and *D*, mouse LFs were transfected with scrambled mimic (10 nm) or miR-98 mimic (10 nm) for 6 h, then treated with or without nicotine (*NIC*, 50 µg/ml) for 72 h. mRNA was isolated for qRT-PCR analysis. Treatment with miR-98 mimic attenuated nicotine (*NIC*)-mediated increases in FN1 (*C*) and ET-1 (*D*) mRNA levels. *Error bars* represent mean ± S.E., *n* = 11, *p < 0.05 *versus* untreated controls (*CON*), ^+^*p* < 0.05 *versus* NIC.

To further explore the role of miR-98 in regulating mediators of airway remodeling, primary mouse LFs in the absence of nicotine were treated with anti-miR-98, and FN1 and ET-1 expression levels were determined. Compared with cells treated with a scrambled anti-miR, treatment with anti-miR-98 recapitulated the nicotine-induced increases in FN1 (Fig. 5*A*) and ET-1 (Fig. 5*B*) mRNA levels. These results demonstrate that reductions in miR-98 levels are sufficient to stimulate expression of markers of airway remodeling. Furthermore, treatment with the miR-98 mimic abrogated nicotine-stimulated increases in FN1 and ET-1, thus demonstrating that miR-98 negatively regulates expression of FN1 (Fig. 5*C*) and ET-1 (Fig. 5*D*).

### Rosiglitazone abrogates nicotine-induced expression of NGF, FN1, and ET-1

**Figure 6:**
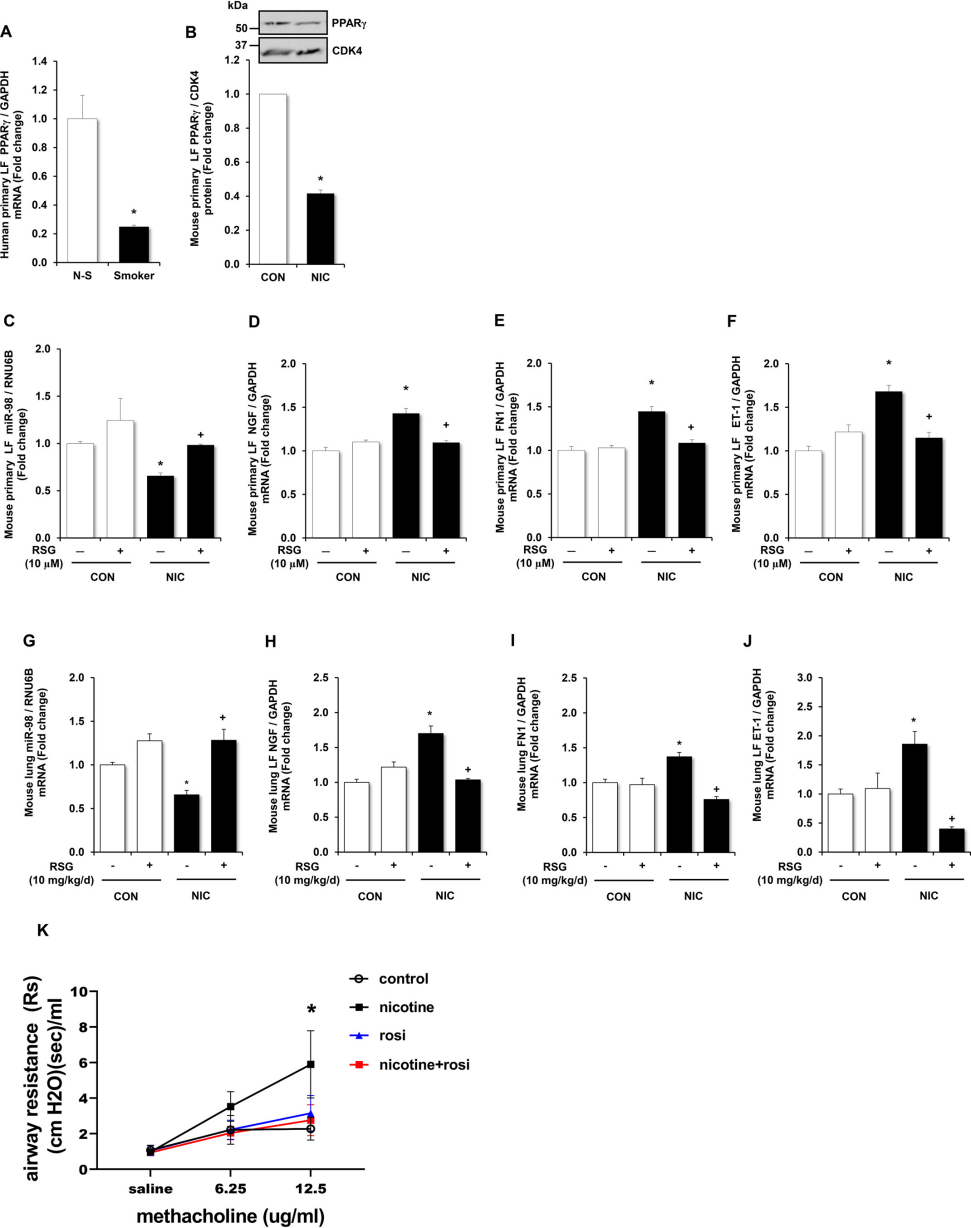
**Rosiglitazone restores miR-98 levels in nicotine-treated fibroblasts and mouse lungs chronically exposed to nicotine, attenuates nicotine-induced expression of NGF, FN1, and ET-1, and attenuates nicotine-induced AHR.***A*, PPARγ mRNA expression was measured by qRT-PCR in primary human lung fibroblasts (Human LF) from smokers (*Smoker*) and nonsmokers (*N-S*). *Error bars* represent mean ± S.E., *n* = 3, *p < 0.05 *versus* nonsmokers (*N-S*). *B*, PPARγ protein levels were measured by Western blotting analysis in mouse LF treated with nicotine (*NIC*, 50 µg/ml) for 72 h. PPARγ protein levels were significantly decreased in nicotine-treated fibroblasts (*NIC*). *Error bars* represent mean ± S.E., *n* = 3, *p < 0.05 *versus* untreated controls (*CON*). *C*–*F*, mouse LFs were treated with nicotine (50 µg/ml) for 72 h, and rosiglitazone (10 µm) was added for the last 24 h. miRNAs were isolated for qRT-PCR analysis of miR-98 (*C*), *error bars* represent mean ± S.E., *n* = 3–9, *p < 0.05 *versus* untreated controls (*CON*), ^+^*p* < 0.05 *versus* NIC. mRNA was isolated for qRT-PCR analysis of NGF (*D*), FN1 (*E*), and ET-1 (*F*). *Error bars* represent mean ± S.E., *n* = 5–10, *p < 0.05 *versus* CON, ^+^*p* < 0.05 *versus* nicotine treated without rosiglitazone. C57BL/6J mice were administered nicotine (100 µg/ml) in the drinking water *ad libitum* for 3 weeks. Selected animals were administered rosiglitazone (20 mg/kg/day) or vehicle by gavage for the last 5 days of nicotine exposure. Animals were anesthetized, tracheostomized, and mechanically ventilated for pulmonary function testing, and then lung tissue was harvested for mRNA isolation for qPCR analysis. In animals exposed to nicotine, rosiglitazone treatment attenuated the nicotine-induced decrease in miR-98 mRNA levels (*G*), and attenuated nicotine-induced increases in NGF (*H*), FN1 (*I*), and ET-1 (*J*). *n* = 5, *p < 0.05 *versus* untreated controls (*CON*), +*p* < 0.05 *versus* NIC. Airway resistance was measured with saline and increasing doses of inhaled methacholine to determine AHR using the flexiVent (EMKA). Rosiglitazone (*RSG*) mitigated the nicotine-induced increase in AHR (*F*). *n* = 5-7, *p < 0.05 nicotine *versus* untreated control and nicotine *versus* nicotine+RSG. *rosi*, rosiglitazone

PPARγ activation improves airway remodeling and airway smooth muscle cell contractility in asthma models (38). Previous studies found that nicotine reduces PPARγ expression in fetal fibroblasts (39). PPARγ knockdown also decreases miR-98 expression in the lung (40). Therefore, to examine the potential role of PPARγ in regulating nicotine-stimulated NGF and miR-98 expression, PPARγ levels were measured in human LFs from nonsmokers and smokers and in murine LFs exposed to nicotine (39). As shown in Fig. 6, *A* and *B*, smoking or nicotine treatment decreased PPARγ mRNA and protein levels. In contrast, treatment with rosiglitazone, an activator of PPARγ pathway, reversed nicotine-mediated decreases in miR-98 levels in murine LFs. However, rosiglitazone itself did not affect the expression of miR-98 (Fig. 6*C*). Interestingly, stimulating PPARγ activity with rosiglitazone abrogated the nicotine-induced increases in NGF (Fig. 6*D*), FN1 (Fig. 6*E*), and ET-1 (Fig. 6*F*) expression in murine LFs, suggesting that PPARγ lies upstream of miR-98. To further examine the relationship between PPARγ and miR-98, murine LFs were treated with anti-miR-98 and rosiglitazone. When compared with untreated murine LFs treated with rosiglitazone alone, anti-miR-98 treatment attenuates the inhibitory effect of rosiglitazone on nicotine-induced ET-1, FN1, COL1A1, and COL3A1 expression (Fig. S8).

To explore whether PPARγ stimulation is a potential intervention to ameliorate nicotine-induced airway remodeling, rosiglitazone was administered during the last 5 days of nicotine exposure to animals chronically exposed to nicotine for 3 weeks. Rosiglitazone increased mir-98 expression in the nicotine-exposed lung and abrogated nicotine-induced increases in NGF, FN1, and ET-1 expression (Fig. 6, *G*–*J*). Similar to our previous findings, animals exposed to nicotine had increased AHR, but rosiglitazone treatment decreased nicotine-induced AHR (Fig. 6*K*) with a trend toward decreased peribronchial collagen deposition with rosiglitazone treatment (Fig. S9). Taken together, these data suggest that stimulating PPARγ activity may be a feasible target for intervention to decrease airway remodeling associated with nicotine and that the mechanism of action depends on miR-98.

## Discussion

**Figure 7:**
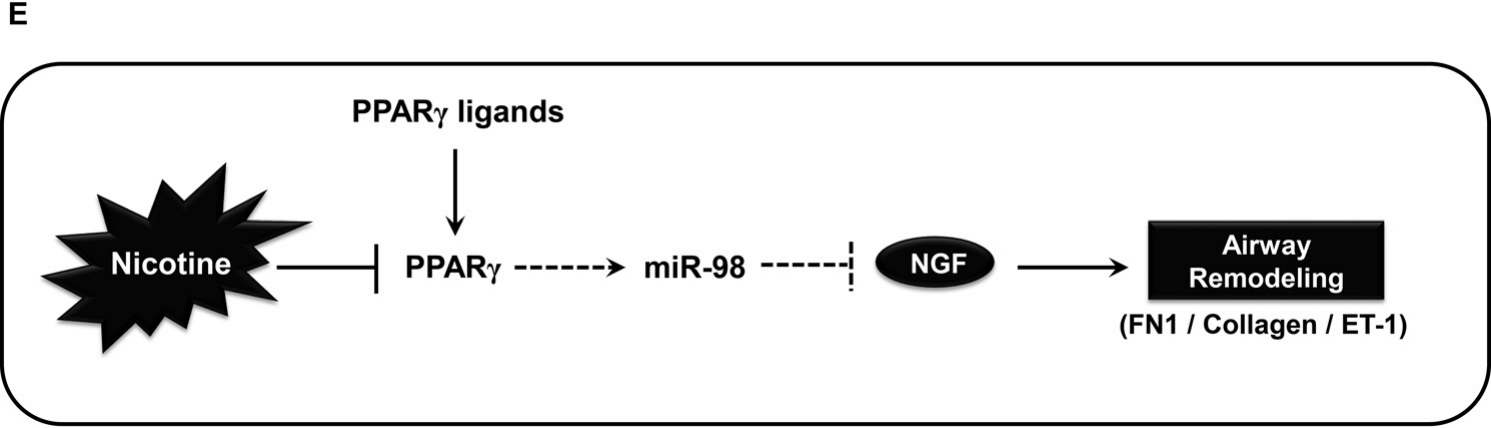
Putative PPARγ-miR-98-NGF axis in nicotine-induced airway remodeling.

These findings illustrate a novel miR-98–dependent mechanism of NGF regulation in lung fibroblasts contributing to airway remodeling (Fig. 7). Airway remodeling is an important histologic characteristic of asthma, and increased remodeling correlates with disease severity (41). In addition, irreversible structural changes from airway remodeling correlate with persistent AHR (42). Therefore, minimizing airway remodeling can potentially modify AHR. It is well-established that cigarette smoke exposure causes airway remodeling in models of allergic asthma and that cigarette smoking decreases the effectiveness of mainstay asthma therapies such as inhaled corticosteroids (43, 44). The current findings demonstrate that chronic exposure to nicotine alone can increase AHR *in vivo* even without allergen sensitization. The absence of allergen sensitization *in vivo* is particularly notable because the increased AHR occurs despite an additional Th2 inflammatory stimulus.

With the increasing popularity of electronic cigarettes (e-cigarettes) and the pervasiveness of cigarette smoking despite aggressive smoking cessation efforts, understanding the effects of nicotine in the lung is becoming increasingly important (45, 46, 47). This study provides additional evidence that nicotine, a major component of cigarette smoke known for its addictive properties, plays a critical role in airway remodeling. Nicotine was previously shown to upregulate expression of FN1 and collagen, both markers of airway remodeling, in LFs through cAMP response element binding protein phosphorylation-dependent pathways and transdifferentiation to myofibroblasts (13, 39). Additionally, chronic exposure to e-cigarette vapor containing nicotine leads to fibrosis in organs other than the lung in animal models (48). The current findings extend this field to demonstrate that 1) increases in FN1 and collagen are also observed in LFs obtained from smokers and 2) miR-98 critically regulates nicotine-stimulated NGF expression in LFs.

Given that airway remodeling and AHR are seen together with chronic nicotine exposure, we sought to determine whether there was a common pathway between the two. NGF, a neurotrophin highly associated with AHR, has also been implicated in other lung diseases in addition to asthma. Monocrotaline-induced pulmonary hypertension is associated with increased NGF protein in lung tissue (49). NGF has also been implicated in viral respiratory infections, either by enhancing viral entry into the cell or by causing airway inflammation (50, 51). Because nicotine stimulated expression of NGF, we then focused on determining whether miR-98 regulated NGF expression (21). miR-98 upregulation has been associated with vascular remodeling in smokers and patients with chronic obstructive pulmonary disease (52). Decreased miR-98 levels have been associated with the development of pulmonary fibrosis in a model of bleomycin-induced lung injury (53). In the current study, we examined human LFs isolated from smokers and nonsmokers and employed two models of nicotine exposure in the airway involving mouse LFs exposed to nicotine *in vitro* and a murine model of chronic nicotine exposure *in vivo* (12, 21, 54). Nicotine or smoke exposure reduced levels of miR-98/let-7 family miRNAs. Our results also demonstrate that nicotine exposure decreases miR-98 and other members of the let-7 family in a dose- and time-dependent manner.

Interestingly, FN1 and ET-1 were also found to be increased with nicotine exposure. FN1 is a commonly used marker for airway remodeling in AHR because myofibroblast differentiation and subepithelial fibrosis are key components of airway remodeling (55, 56, 57). In the ovalbumin allergic asthma model, mice lacking the extra domain A of fibronectin have decreased AHR and allergic inflammation when compared with WT mice (10). A recent study demonstrates that ET-1 is the predominant endothelin in human LFs and induces collagen synthesis (58). Furthermore, ET-1 contributes to airway remodeling by modulating transforming growth factor β/SMAD-driven peribronchial collagen deposition (32). Our results demonstrate that treatment with anti-miR-98 increased expression of NGF, FN1, and ET-1. Conversely, treatment with a miR-98 mimic abrogated the nicotine-induced increases in NGF, FN1, and ET-1 expression. These findings confirm that miR-98 plays an important role not only in regulating nicotine-stimulated NGF but also in expression of other possible mediators of airway remodeling, such as FN1, and ET-1 in LFs. Furthermore, our data show that nicotine-stimulated FN1 and ET-1 expression is NGF-dependent.

NGF regulation by the let-7 family of miRNA has been previously reported. Let-7 family members are known to target NGF in nerve regeneration models. In a rat model of sciatic nerve injury, reduced expression of let-7 family members was associated with increased NGF mRNA and protein levels and enhanced nerve regeneration, although nerve injury alone did not affect let-7 family members equally (59). More recently, increased miR-98 expression has been associated with decreased NGF expression in human coronary endothelial cells exposed to hypoxia/reoxygenation. Antagonizing miR-98 in a rat ischemia/reperfusion model increased NGF expression in the heart and improved microvascular dysfunction (60). These studies suggest that other let-7 family members could potentially regulate NGF in our model and contribute to nicotine-induced airway remodeling. Additional studies are needed to explore this possibility to delineate whether miR-98 is the predominant miRNA involved in NGF-mediated airway remodeling.

Previous reports have suggested that activation of the nuclear hormone receptor PPARγ may regulate inflammation, airway smooth muscle contractile function, and airway remodeling to reduce AHR (38, 61, 62). Fitting with these reports, we found that nicotine decreased PPARγ expression in mouse LF and that PPARγ expression was reduced in LFs isolated from smokers. These findings are concordant with studies showing that maternal perinatal nicotine exposure reduced PPARγ expression in the lungs of offspring (36, 63). In addition, recent studies show that PPARγ activity regulates expression of miR-98, and in turn, miR-98 negatively regulates ET-1 expression in hypoxia-induced pulmonary hypertension (64).

Because our results demonstrated that nicotine exposure similarly reduced miR-98 levels and increased ET-1 expression, we examined whether modulating PPARγ activity would modify nicotine-induced reductions in miR-98 and markers of airway remodeling. Our findings confirm that PPARγ activation with rosiglitazone attenuated nicotine-induced expression of NGF, FN1, and ET-1 in LFs and restored miR-98 to control levels. Taken together, these data support a paradigm where nicotine decreases PPARγ, leading to decreased miR-98 expression and increased NGF, FN1, and ET-1 expression (Fig. 7). Thus, increasing PPARγ activity will result in increased miR-98 expression and decreased expression of NGF, FN1, and ET-1 with presumably less airway remodeling after nicotine exposure. Our data are consistent with prior literature showing that administration of PPARγ ligands improves airway remodeling and airway smooth muscle contractility in models of allergic asthma (38). Because our study included only one rosiglitazone treatment regimen, additional studies optimizing the rosiglitazone dosing strategy are needed to further examine our findings that rosiglitazone decreases nicotine-induced peribronchial collagen deposition. Similar to our animal data demonstrating that rosiglitazone administration decreased nicotine-induced AHR, a small clinical study also found that rosiglitazone administration significantly improved pulmonary function parameters when compared with inhaled beclomethasone alone in smoking asthmatics (62). By increasing miR-98 levels, PPARγ ligands may be a viable therapy to lessen airway remodeling and subsequent AHR in asthmatics exposed to nicotine through cigarette smoke, nicotine replacement therapy, or e-cigarette use.

Although the current study focuses on nicotine as a key smoking-related mediator, cigarette smoke contains many more compounds than just nicotine that could augment or diminish the effects of nicotine (65, 66). In addition, the duration of nicotine exposure and age during exposure are important variables that should be considered when determining the effects of nicotine (67). However, data in the current study from human LFs isolated from smokers and nonsmokers describe findings similar in magnitude to those seen in fibroblasts and whole lung homogenates exposed to nicotine alone. These findings suggest that a significant portion of the effects of cigarette smoke on airway remodeling are attributable to nicotine. Such findings are particularly relevant with the increasing number of people using e-cigarette liquid containing nicotine as a substitute for combustible tobacco, including both youths and adults (68, 69). Furthermore, the current findings regarding the effects of nicotine alone may have additional clinical implications for patients exposed to nicotine from other sources, such as electronic cigarettes.

Because of the important role fibroblasts play in airway remodeling, the relative contribution of fibroblasts to AHR requires further study. The pathophysiology of AHR is complex, and a majority of studies have focused on the contribution of immune cells and airway SMCs to AHR. Other cell types can also be stimulated by nicotine to increase NGF expression and thus can also play a role in the pathogenesis of nicotine-promoted AHR (Fig. S2). Currently, there is little known about miR-98 regulation of NGF in other cells types. Fibroblasts play an integral role in airway remodeling, although their contribution to AHR pathogenesis is still emerging. The results presented do not address the additional influence of immune cells and airway smooth muscle cells on fibroblast-mediated airway remodeling and AHR, but investigations into their interactions are ongoing. Nonetheless, the current findings delineate a clear miR-98–dependent pathway that underlies nicotine-induced airway remodeling and reveal that targeting PPARγ activity *in vivo* improves AHR and airway remodeling (42, 66).

In summary, the current study clarifies a novel pathway whereby nicotine exposure reduces miR-98 levels that negatively regulate NGF, FN1, and ET-1 expression. Our findings suggest miR-98 as a potential therapeutic target to modulate nicotine-associated airway remodeling. Modulating PPARγ activity is another viable therapeutic approach to increase miR-98 expression and subsequently decrease the airway remodeling seen after nicotine exposure. Because strategies to modify airway remodeling and improve asthma control are still emerging, these results have implications for patients with lung disease associated with increased NGF, such as asthma, who are also exposed to cigarette smoke (voluntarily or involuntarily) or to nicotine-containing products. Even though cigarette smoking rates have declined over the past decades, 25% of current asthmatics are smokers. Cigarette smoke exposure remains an environmental trigger for asthma symptoms and poor control for asthmatic patients partially through corticosteroid resistance. Therefore, identifying alternative therapeutic approaches to corticosteroids is essential (44). The growing popularity of electronic cigarettes will likely increase the prevalence of nicotine exposure as well. Lastly, these results add to the growing body of knowledge indicating that the fibroblast plays an important role in AHR through airway remodeling and that studying alternative approaches to reduce airway remodeling should continue.

## Experimental procedures

### Reagents

NGF, ET-1, FN1, PPARγ, CDK4, and GAPDH antibodies were obtained from Santa Cruz Biotechnology. The following reagents were supplied by Sigma-Aldrich: FBS and DMSO. Human scrambled siRNA, mimic miR-98, and anti-miR-98 were purchased from Qiagen. The PPARγ ligand rosiglitazone was purchased from Cayman Chemical Company.

### Chronic nicotine-fed mouse model with rosiglitazone administration

Animal care, handling, and experimental procedures followed a protocol approved by the Atlanta VA Medical Center Institutional Animal Care and Use Committee. 8–10-week-old C57BL/6J male mice were administered nicotine (100 μg/ml, Sigma-Aldrich) in the drinking water or untreated water for 4 weeks. This technique generates serum nicotine levels in mice comparable with those reported in smokers as previously described (34, 67). Selected animals were gavaged daily with 100 μl of rosiglitazone suspended in 0.5% methyl cellulose at 20 mg/kg/day or with an equivalent volume of vehicle for the last 5 days in a 3 week nicotine exposure (70). Lungs were harvested after pulmonary function measurement for RNA and protein isolation.

### Pulmonary function measurement with methacholine challenge

Respiratory mechanics were measured with a forced oscillation technique by fitting the constant phase model using the integrated software in the flexiVent System (EMKA, Montreal, Canada) in anesthetized, tracheostomized, 12–14-week-old male mice at the completion of nicotine treatment (12). Increasing doses of inhaled methacholine (6.25–100 mg/ml) were administered via nebulization, and three consecutive peak airway resistance measurements were recorded after each dose. Coefficient of determination for recorded measurements was >95%.

### Mouse primary lung fibroblasts

Mouse primary LFs were harvested and cultured from 8–12-week-old C57BL/6J male mice as previously described (21). In selected studies, mouse LFs were treated with nicotine (NIC, 50 μg/ml) or an equivalent volume of culture medium (CON) for 72 h, and rosiglitazone (10 μm) or an equivalent volume of vehicle was added for the last 24 h.

### Human primary lung fibroblasts

Human LFs were isolated from de-identified human lungs not suitable for transplantation and donated for medical research from the National Disease Research Interchange (Philadelphia, PA, USA) and the International Institute for the Advancement of Medicine (Edison, NJ, USA) as previously described (54). Donors were without a history of chronic lung disease and with reasonable lung function with a PaO_2_/FIO_2_ ratio >225, a clinical history and X-ray that did not indicate infection, and limited time on a ventilator. Smokers were individuals who smoked 10–25 cigarettes per day for at least 3 years, and nonsmokers included those who had never smoked. Because the lungs were from de-identified organ donors, these studies were approved as exempt research by the Committee for the Protection of Human Subjects at National Jewish Health and the Atlanta VA Healthcare System. LFs were grown in DMEM supplemented with 10% FBS.

### PicroSirius Red staining

Lungs were harvested from euthanized mice fed nicotine (100 μg/ml) in the drinking water or from mice not exposed to nicotine. The lungs were then inflated to 20 cm H_2_O with 4% paraformaldehyde, then dehydrated and embedded in paraffin as previously described (12). To examine peribronchial collagen I and III deposition, 5-μm sections of paraffin-embedded lungs were stained with PicroSirius Red (ScyTek Laboratories, Logan, UT) according to manufacturer's protocol. Three random lung sections per mouse were examined, and representative images were included in the results.

### NGF 3′UTR luciferase reporter studies

The pLightSwitch-empty-vector and pLightSwitch-NGF-3′UTR construct containing the 3′UTR of mouse NGF (NM_001112698), the putative binding site for miR-98, and mutant NGF-3′UTR construct (5′-CTTGCCTGCAGCCCCCTTCCCCACCTGCCCCCTCCACACTCTCCTGGGCCCCTCC**CATCGAC**AGCCTGTAAATTATTTTAAATTATAAGGACTGCATGATAATTTATCGTTTATACAATTTTA-3′) were purchased from SwitchGear Genomics (Carlsbad, CA). Bold letters denote the miR-98 seed match sequence, with the mutated bases of the miR-98 seed match underlined. Mouse LFs (1 × 10^4^) were seeded into 24-well plates, incubated for 24 h, and washed with PBS, and then fresh growth medium was added before addition of transfection complexes. For luciferase assays, 200 ng of pLightSwitch-empty-vector, NGF-3′UTR, or mutant NGF-3′UTR construct with 10–25 nm miR-98 mimic or scrambled siRNA were transiently co-transfected into LFs using lipofectamine RNAiMax (Invitrogen) according to manufacturer's instructions. After co-transfection for 6 h, the transfection media were replaced by DMEM containing 10% FBS. Mouse LFs were cultured in fresh media for 72 h. LF lysates were harvested and analyzed for luciferase activity using the Luciferase Reporter Assay System (SwitchGear Genomics), and luciferase activity was measured using a Luminometer (PerkinElmer). Relative light units were normalized to control group luciferase activity.

### mRNA stability assay

To inhibit *de novo* NGF mRNA synthesis, mouse primary LFs were transfected with scrambled RNA (SCR) or mimic miR-98 for 72 h, and then 5 μg/ml actinomycin D (Apexbio, Houston, TX) was treated time-dependently. Total RNAs were isolated using mirVana kit (Thermo Fisher Scientific), and NGF mRNA levels were measured by qRT-PCR analysis. NGF mRNA *t*_1/2_ was determined by comparing to the mRNA level before adding actinomycin D.

### miR-98 down-regulation and overexpression

To confirm the role of miR-98 in alterations in NGF expression, LFs were transfected with lipofectamine RNAiMax containing anti-miR-98 (50 nm), anti-miR negative control (50 nm), mimic miR-98 (10 nm), or scrambled mimic (10 nm) according to the manufacturer's (Qiagen) instructions as previously reported (71). Six hours after transfection, serum-free medium was replaced with 10% FBS DMEM. Mouse LFs were treated with control or nicotine for 24, 48, or 72 h as we previously reported, and then alterations in miR-98, NGF, ET-1, PPARγ, FN1, and collagen levels were confirmed by qRT-PCR (72). The relative abundance of these targets was reflected by the mean number of cycles of qRT-PCR amplification required for their detection (mean Ct values: miR-98 = 24.2 and RNU6B = 16.8 in mouse LFs, miR-98 = 25.3 and RNU6B = 17.8 in mouse lungs, and miR-98 = 23.5 and RNU6B = 18.0 in human LFs).

### mRNA and miRNA analysis

To measure miR-98, NGF, ET-1, PPARγ, FN1, and collagen levels in human and mouse LFs or mouse lungs, small RNAs (<200 nucleotides) and large RNAs (>200 nucleotides) were isolated using the mirVana kit (Invitrogen). The levels of miR-98/let-7 family were analyzed by qRT-PCR using Qiagen miRNA primer assay (Qiagen) according to the manufacturer's instructions. RNU6B was used as a control for miRNA levels. NGF, ET-1, PPARγ, FN1, collagen type 1A (COL1A1), and collagen type 3A (COL3A1) mRNA levels in the same sample were determined and quantified using specific mRNA primers as previously described (72). GAPDH mRNA was used as a control.

### NGF loss-of-function

For NGF loss-of-function, mouse primary LFs were transfected with scrambled or NGF RNAi duplexes (100 nm, Integrated DNA Technologies, Coralville, IA) using Lipofectamine 3000 transfection reagent (Invitrogen) according to the manufacturer's instructions. After transfection for 6 h, the transfection media were replaced with DMEM containing 5% FBS and incubated at room temperature for 72 h. Mouse LF lysates were then harvested and examined for NGF, FN1, ET-1 COL1A1, COL3A1, and 9S levels using qRT-PCR analysis.

### RIP assay

RIP assay was performed using the Magna RIP RNA-binding protein immunoprecipitation kit (MilliporeSigma, Burlington, MA) according to the manufacturer's instructions. Briefly, mouse LFs were lysed in 100 μl of RIP lysis buffer. The whole LF extract was incubated with anti-argonaute 2 (Ago2) antibody (Abcam) or negative control (mouse IgG, Abcam) in RIP buffer containing protein A/G magnetic beads. After washing to remove unbound materials, RNAs were extracted from the magnetic bead-bound complexes. The miR-98 levels in the precipitates were determined by qRT-PCR analysis.

### Western blotting analysis

Mouse lung or LF protein lysates were subjected to Western blotting analysis as we reported (72). Primary antibodies included NGF (1:250), ET-1 (1:500), PPARγ (1:500), FN1 (1:500), GAPDH (1:2000), and CDK4 (1:2000) antibodies. Proteins were visualized using a peroxidase-coupled anti-goat or anti-rabbit IgG in the presence of LumiGlo reagent (Thermo Fisher Scientific). Bands were identified by chemiluminescence, quantified by laser densitometry (Bio-Rad), and normalized to GAPDH or CDK4 levels within the same lane.

### Statistical analysis

Data from studies with more than two groups were analyzed using analysis of variance. Post-hoc analysis used the Student Neuman Keuls and Tukey's test to detect differences between specific groups. In studies comparing only two experimental groups, data were analyzed with Student's *t* test to determine the significance of treatment effects. The level of statistical significance was taken as *p* < 0.05. All results are reported as mean ± S.E.

## Data availability

All the data described in the manuscript are located in this article and in the supporting information.

10.13039/100000050HHS | NIH | National Heart, Lung, and Blood Institute (NHLBI) (HL133053) to Bum-Yong Kang
10.13039/100000050HHS | NIH | National Heart, Lung, and Blood Institute (NHLBI) (HL102167) to C. Michael Hart
10.13039/100000050HHS | NIH | National Heart, Lung, and Blood Institute (NHLBI) (HL118171) to Beata Kosmider
10.13039/100000050HHS | NIH | National Heart, Lung, and Blood Institute (NHLBI) (HL130230) to Qing Lu
10.13039/100000738U.S. Department of Veterans Affairs (VA) (BX001306) to Cherry Wongtrakool
10.13039/100000738U.S. Department of Veterans Affairs (VA) (BX001910) to C. Michael Hart
10.13039/100000968American Heart Association (AHA) (13SDG14150004) to Bum-Yong Kang
10.13039/100005640Flight Attendant Medical Research Institute (FAMRI) (130046) to Beata Kosmider
10.13039/100000050HHS | NIH | National Heart, Lung, and Blood Institute (NHLBI) (HL119291) to Changwon Park
