# Supplementary material for: MicroRNA-98 reduces nerve growth factor expression in nicotine-induced airway remodeling
Source: J Biol Chem. 2025 Jan 13;295(52):18051–64. doi: 10.1074/jbc.RA119.012019 (PMC11843582; doi:10.1074/jbc.RA119.012019)
Supplement: Supplementary file 1 [file mmc1.pdf]

# Supplementary Figures

# MicroRNA-98 Reduces Nerve Growth Factor in Nicotine-Induced Airway Remodeling

Cherry Wongtrakool, MD[1], Junsuk Ko, PhD[2], Andrew J. Jang, PhD[3], Kora Grooms, BS[1], Sarah Chang, BS[1], Cory Sylber BS[1], Beata Kosmider, PhD[4], Karim Bahmed, PhD[4], Michael Blackburn[2], PhD[2], Roy L. Sutliff, PhD[1], C. Michael Hart, MD[1], Changwon Park PhD[5], Toru Nyunoya, PhD[6], Michael J. Passineau, PhD[3], Qing Lu, PhD[7], and Bum-Yong Kang, PhD,[1*]

**Supplementary Figure S1**

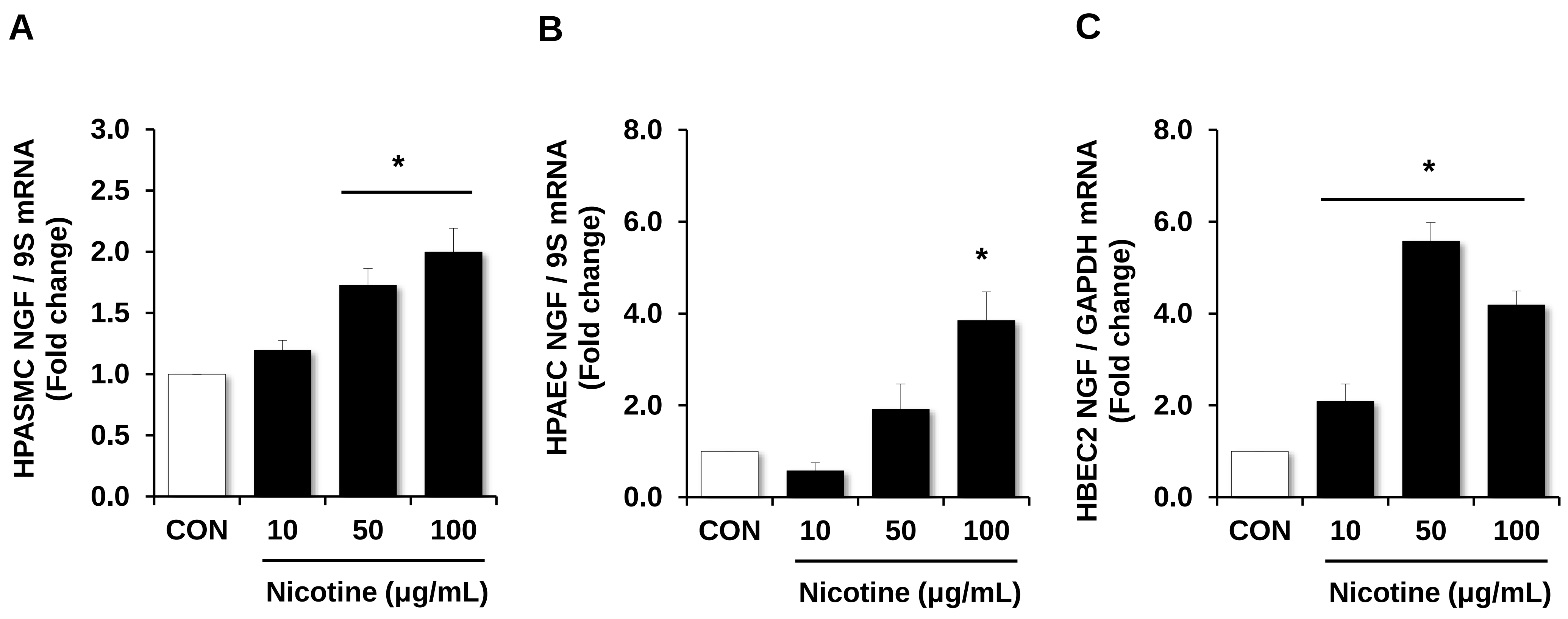


**Supplementary Figure S1. Nicotine exposure increases NGF in several lung cell types** Human pulmonary artery smooth muscle cells (HPASMC), human pulmonary artery endothelial cells (HPAEC), and human bronchial epithelial cells (HBEC2) were cultured and treated with nicotine (NIC, 50 µg/ml) for 72 hours. mRNA was isolated for qRT-PCR analysis. Nicotine significantly increased NGF in all cell types tested. Error bars represent mean ± standard error (SE), n=4-5, *p<0.05 vs. untreated controls (CON).

Supplementary Figure S2

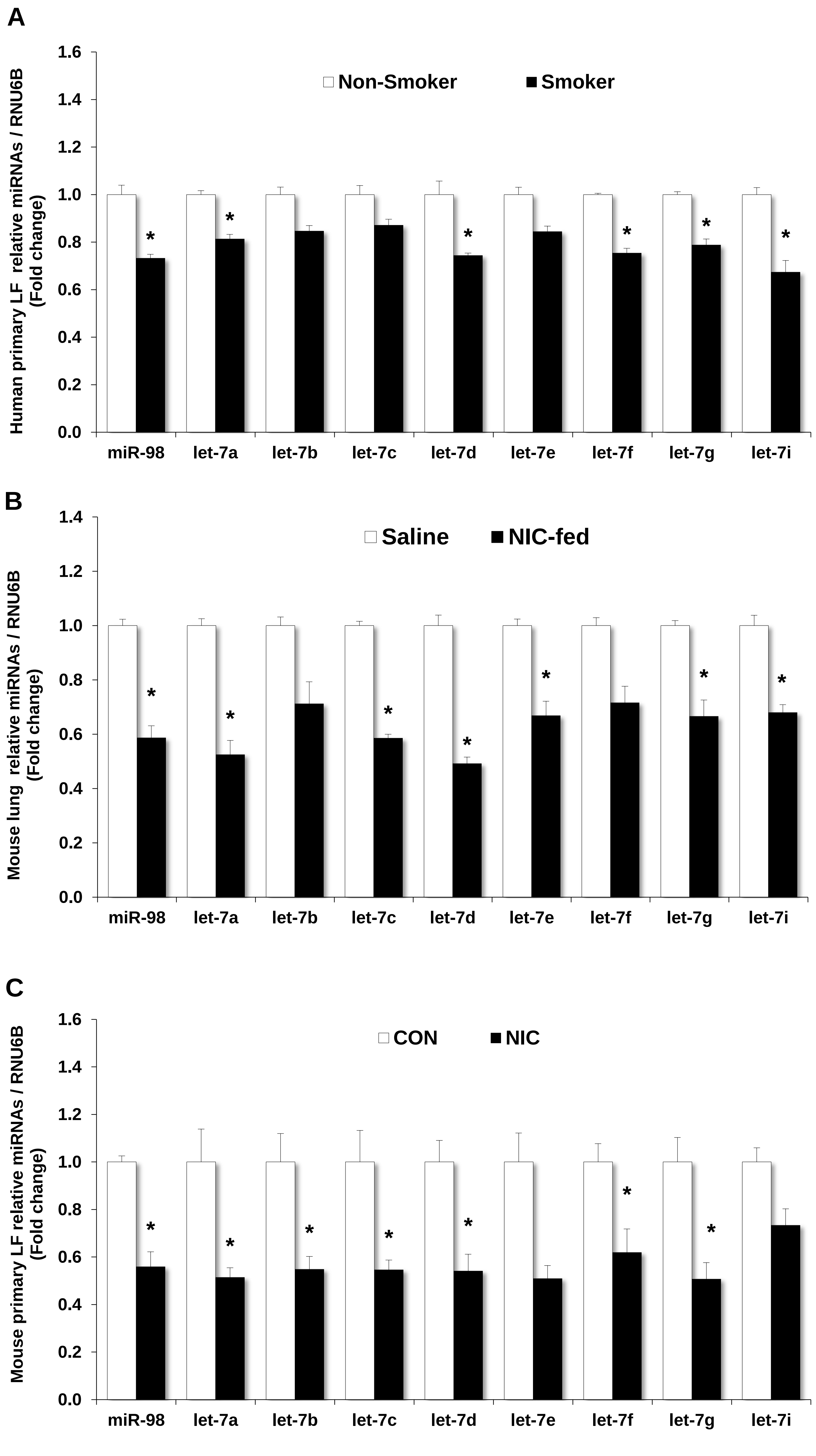


**Supplementary Figure S2. Nicotine exposure decreases expression of miRNAs in the miR-98/let-7 family.** miRNAs were isolated from primary human lung fibroblasts (1LFs) obtained from smoking (Smoker) and non-smoking (Non-smoker) lung donors (**A**), whole lung homogenates obtained from mice chronically exposed to nicotine (NIC, 100 µg/ml) or saline (Saline) in the drinking water for 4 weeks (**B**), and mouse 1LFs were treated with nicotine (NIC, 50 µg/ml) or without NIC (CON) for 72 hours (**C**) for analysis by qRT-PCR. Nicotine exposure alone or cigarette smoke significantly decreases expression of members of the miR-98/let-7 family. Error bars represent mean ± standard error (SE), n=3-6, *p<0.05 vs. controls (non-smoker, Saline, or CON, respectively).

**Supplementary Figure S3**

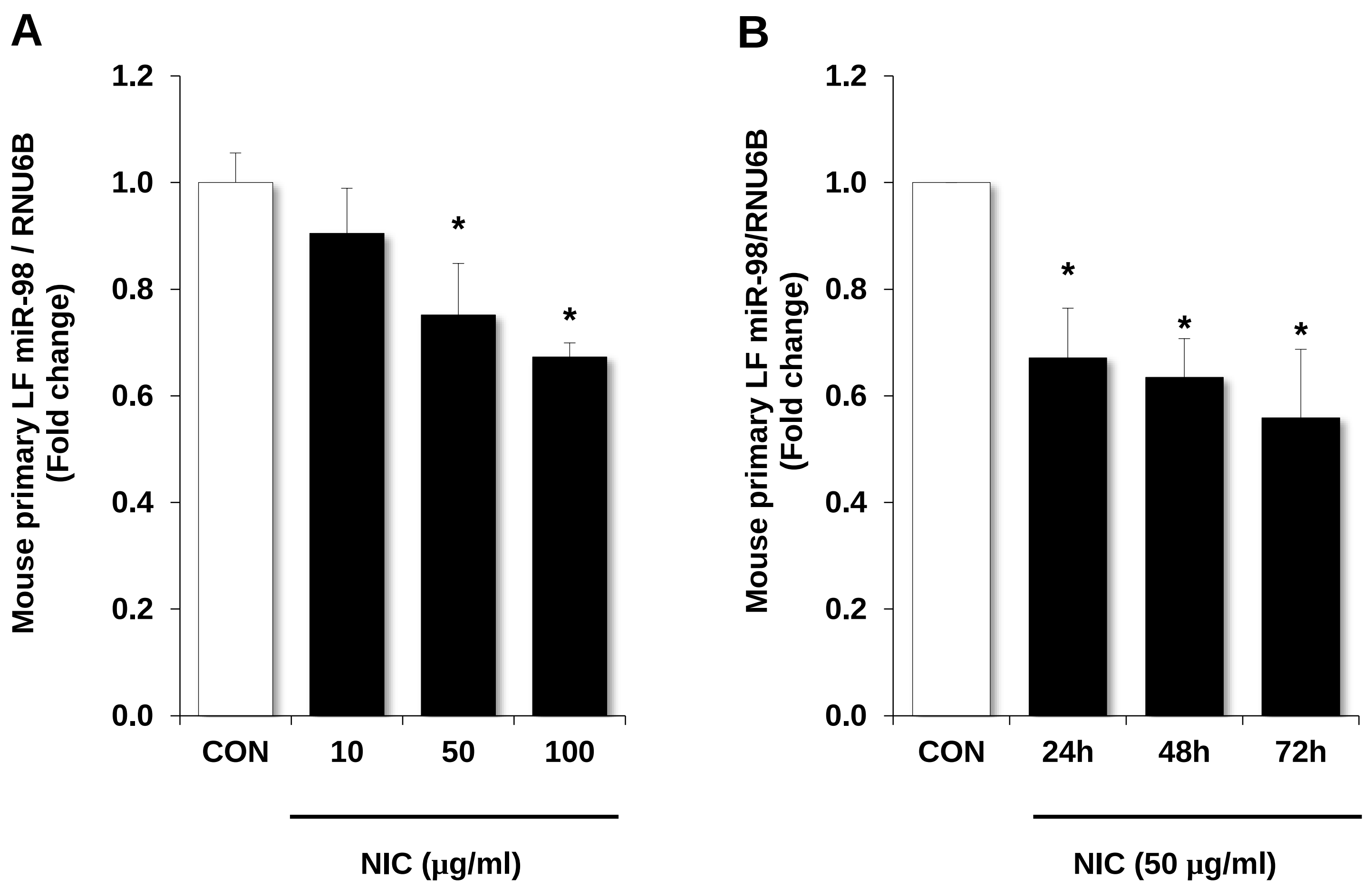


**Supplementary Figure S3. Nicotine exposure reduces miR-98 in a dose and time-dependent manner.** Primary mouse lung fibroblasts (1LFs) were cultured and treated with nicotine (NIC, 50 µg/ml) for 24-72 hours. miRNA and mRNA were isolated for qRT-PCR analysis. Nicotine significantly decreased miR-98 expression in a dose **(A)** and time **(B)** -dependent manner. Error bars represent mean ± standard error (SE), n=5-6, *p<0.05 vs. untreated controls (CON).

## Supplementary Figure S4

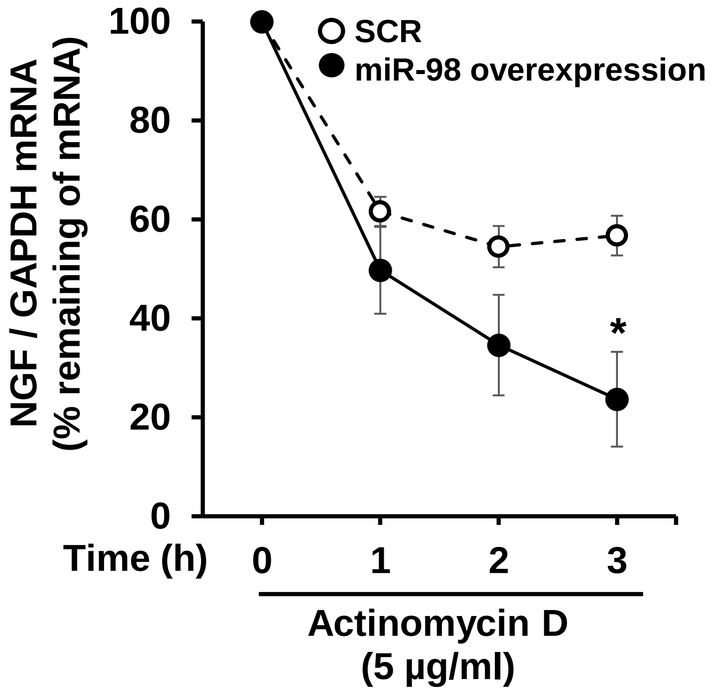


**Supplementary Figure S4. NGF mRNA stability is reduced with miR-98 overexpression.** To inhibit de novo NGF mRNA synthesis, mouse primary LFs were transfected with scrambled or mimic miR-98 for 72 hours, and then were treated with 5 µg/ml actinomycin D (in a time-dependent manner. Total RNAs were isolated to measure NGF mRNA levels by qRT-PCR analysis. NGF mRNA half-life was determined by comparing to the mRNA level before adding actinomycin D. Error bars represent mean ± standard error (SE), n=3, *$p<0.05$ vs. scrambled controls (SCR).

## Supplementary Figure S5

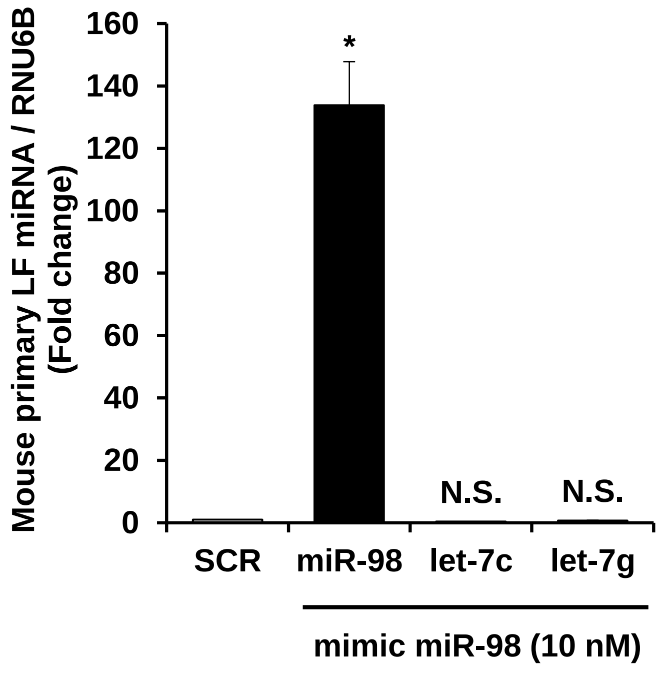


**Supplementary Figure S5. Transfection with miR-98 mimic specifically increases miR-98 levels, but not other members of the let-7 family.** Mouse 1LFs were treated with either scrambled miR (SCR) or 10 nM anti-miR-98 for 6 hours. Mouse 1LFs were then collected, and mRNA were isolated and subjected to qRT-PCR analysis for miR-98 and RNU6B. Each bar represents the mean ± SE ), n=3, *p<0.05 vs. scrambled control (SCR).

## Supplementary Figure S6

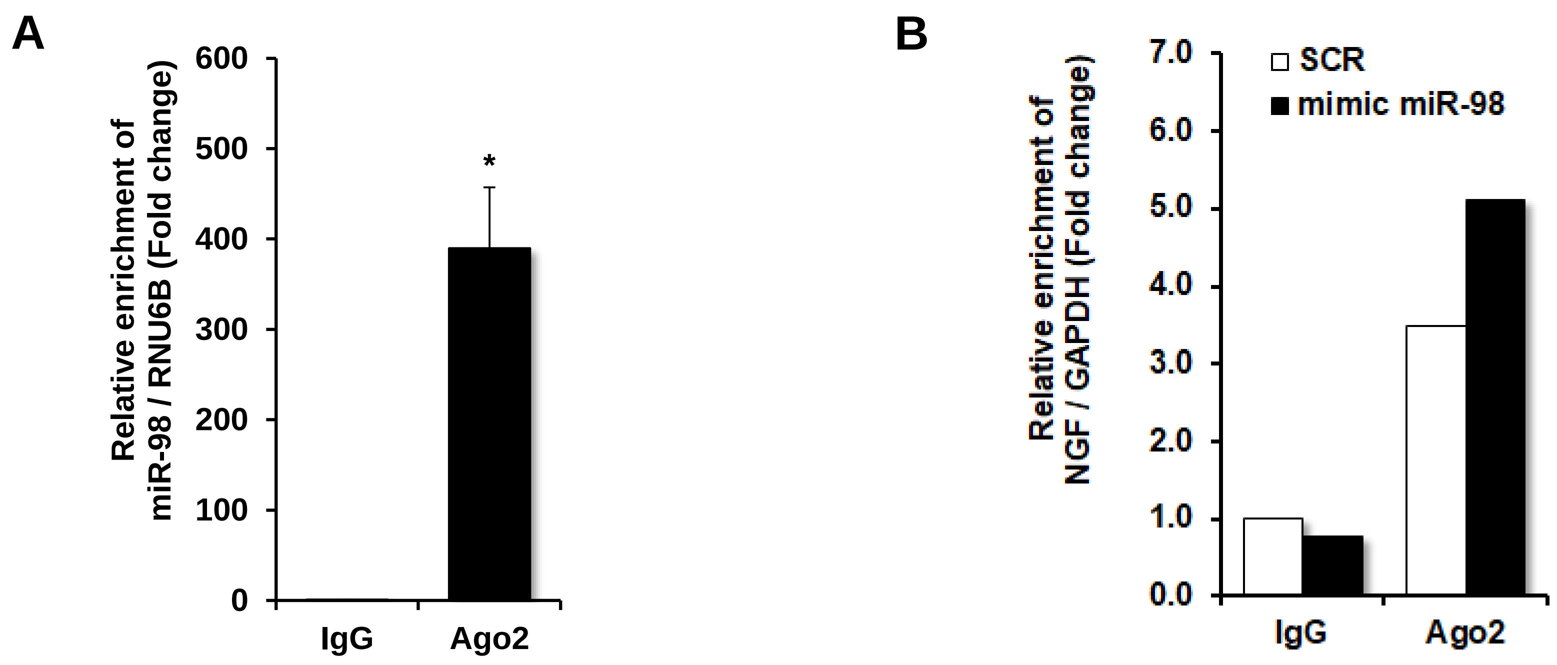


**Supplementary Figure S6. miR-98 is enriched in Ago2 immunoprecipitates in mouse LFs.** Mouse lung fibroblasts (LFs) were lysed in 100 µl RIP lysis buffer. The whole LF extract was incubated with anti-argonaute 2 (Ago2) antibody (Abcam,) or negative control (mouse IgG, Abcam) in RIP buffer containing protein A/G magnetic beads. RNAs were extracted from the magnetic beads bound complexes for qRT-PCR analysis. miR-98 mRNA was significantly enriched in the Ago2 immunoprecipitates compared of immunoprecipitates using an IgG input control (**A**). n=3, *p<0.05 vs. scrambled control (SCR). IMouse LFs transfected with miR-98 mimic prior to Ago2 immunoprecipitation were enriched in NGF mRNA compare to mouse LFs treated with a scrambled miR and IgG immunoprecipitates (**B**). n=1.

**Supplementary Figure  S7**

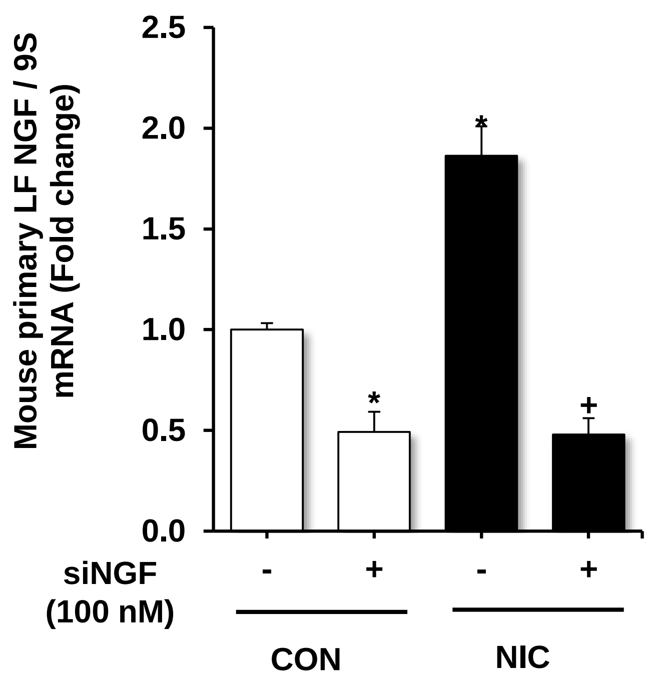


**Supplementary Figure S7. SiRNA knockdown of NGF in mouse lung fibroblasts (LFs).** Mouse LFs were transfected with 100 nM siNGF for 6 hours. Mouse LFs were then collected, and mRNA were isolated and subjected to qRT-PCR analysis for NGF and 9S. Each bar represents the mean ± SE ), n=3, *p<0.05 vs. scrambled control (SCR).

**Supplementary Figure S8**

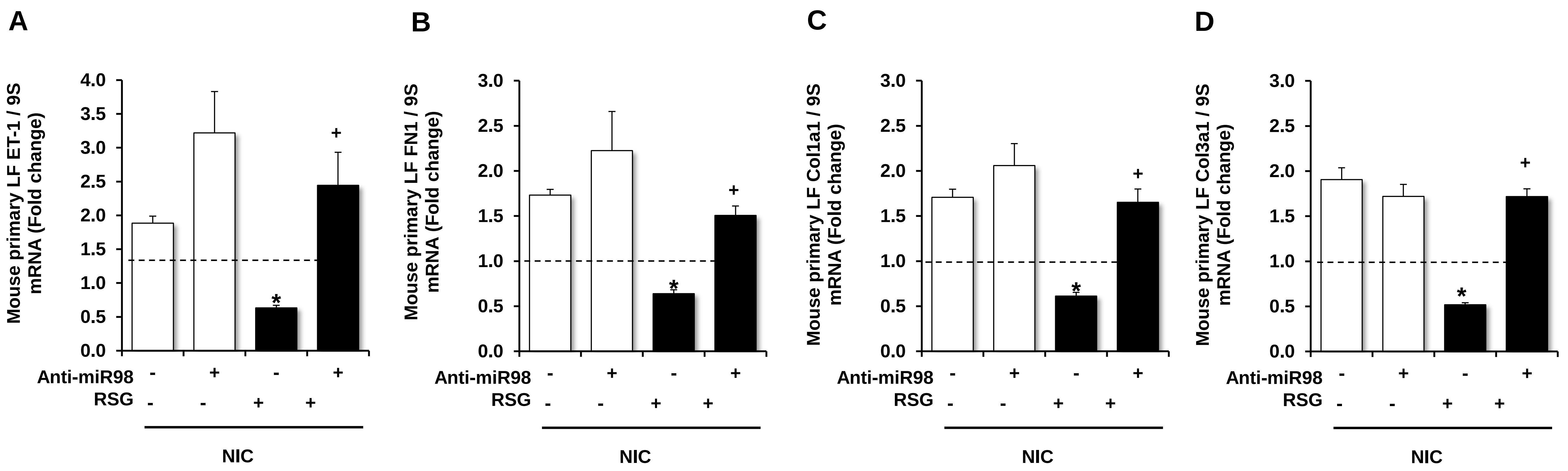


**Supplementary Figure S8. Anti-miR-98 attentuates the inhibitory effect of rosiglitazone in mouse lung fibroblasts (LFs).** Mouse LFs were transfected with either scrambled miR (SCR) or 50 nM anti-miR-98 for 6 hours. Mouse LFs were then treated with nicotine (50 µg/ml) for 72 hours and rosiglitazone (10 µM, RSG) was added for the last 24 hours. mRNA were isolated and subjected to qRT-PCR analysis. The presence of anti-miR-98 in nicotine-exposed cells treated with rosiglitazone attenuates nicotine-induced decreases in ET-1 (**A**), FN1 (**B**), COL1A1 (**C**), and COL3A1 (**D**) expression levels. Each bar represents the mean ± SE ), n=3, *p<0.05 vs. control LFs treated with scrambled miR and rosiglitazone. Dotted line is the fold change of control LFs treated with scrambled miR and rosiglitazone compared to scrambled miR alone.

**Supplementary Figure S9**

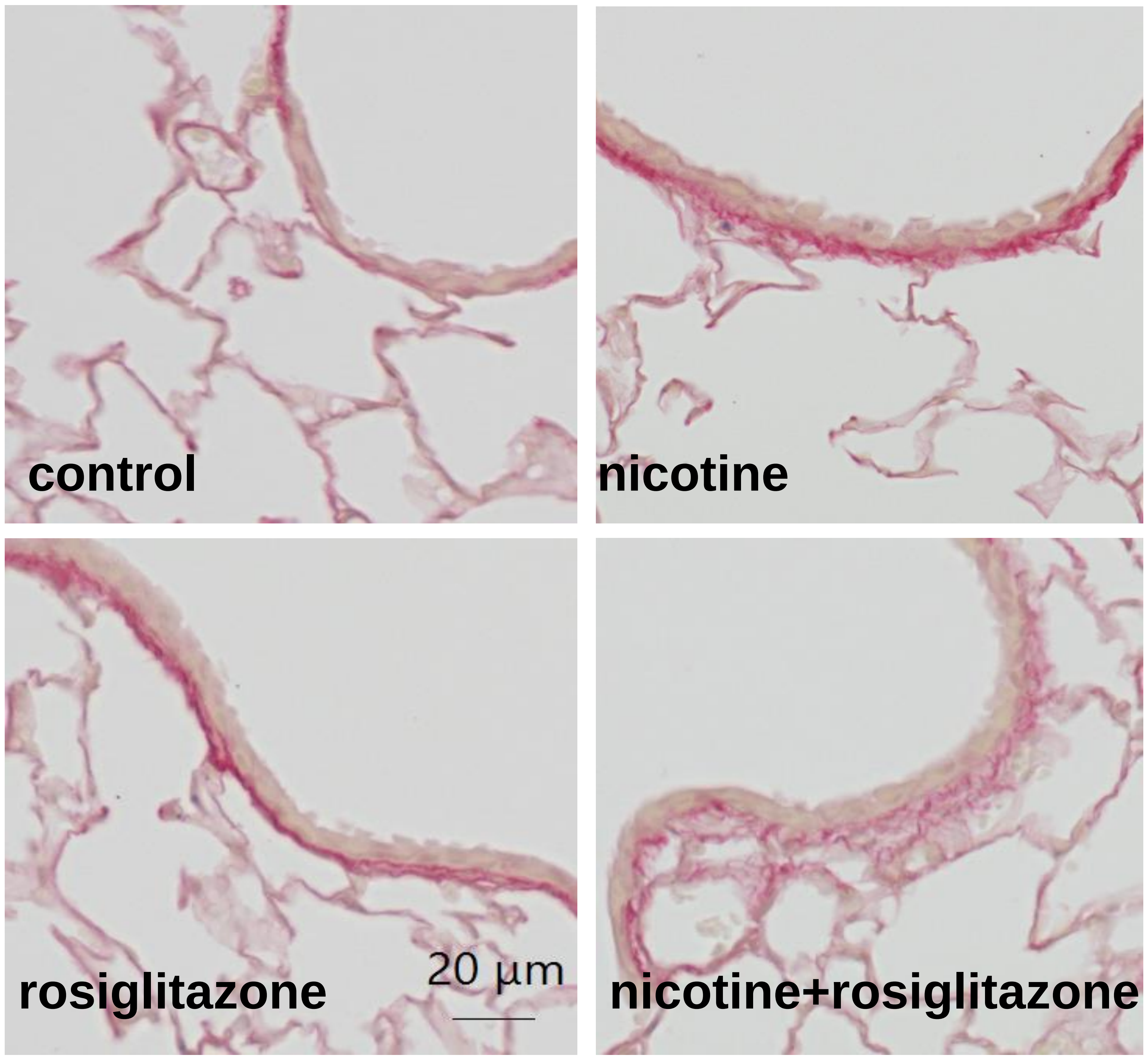


**Supplementary Figure S9. Representative images of Picro-Sirius Red staining of peribronchial collagen.** C57BL/6 mice were administered nicotine (100 μg/ml) in the drinking water for 3 weeks. Selected animals were administered rosiglitazone (20 mg/kg/day) or vehicle by gavage for the last 5 days of nicotine exposure. Lung tissue was harvested, fixed in formalin, and paraffin embedded for Picro-Sirius Red staining of collagen fibers. Animals treated with nicotine exhibit increased peribronchial collagen deposition, and subsequent treatment with rosiglitazone partially decreases sub-epithelial collagen deposition around airways.
